# First study conducted in Northern India that identifies group C rotavirus as the etiological agent of severe diarrhea in children in Delhi

**DOI:** 10.1186/s12985-017-0767-8

**Published:** 2017-05-30

**Authors:** Vasundhara Razdan Tiku, Baoming Jiang, Praveen Kumar, Satender Aneja, Arvind Bagga, Maharaj Kishen Bhan, Pratima Ray

**Affiliations:** 1 Department of Biotechnology, Faculty of Science, Jamia Hamdard University, Hamdard Nagar, New Delhi, 110062 India; 2 Department of Pediatrics, All India Institute of Medical Sciences, New Delhi, India; 3 Centers for Disease Control and Prevention, Atlanta, USA; 4 Kalawati Saran Children’s Hospital, Lady Hardinge Medical College, New Delhi, India

**Keywords:** Diarrhea, Children, Group C Rotavirus, Gastroenteritis, Phylogenetic analysis, Sequence identity matrix

## Abstract

**Background:**

Group C Rotavirus (RVC) is an enteric pathogen responsible for acute gastroenteritis in children and adults globally. At present there are no surveillance studies on group C Rotaviruses in India and therefore their prevalence in India remains unknown. The present study aimed to evaluate group C rotavirus infection among <5 years old children hospitalized with acute gastroenteritis in New Delhi.

**Methods:**

A total of 350 fecal specimens were collected during September 2013 to November 2014 from <5 years old diarrheal patients admitted at KSCH hospital, Delhi. The samples found negative for group A rotavirus (*N* = 180) by Enzyme immunoassay were screened for group C rotavirus by RT-PCR with VP6, VP7 and VP4 gene specific primers. The PCR products were further sequenced (VP6, VP7, VP4) and analyzed to ascertain their origin and G and P genotypes.

**Results:**

Six out of 180 (group A rotavirus negative) samples were found positive for group C rotavirus by VP6 gene specific RT-PCR, of which 3 were also found positive for VP7 and VP4 genes. Phylogenetic analysis of VP7 and VP4 genes of these showed them to be G4 and P[2] genotypes. Overall, the nucleotide sequence data (VP6, VP7 and VP4) revealed a close relationship with the human group C rotavirus with no evidence of animal ancestry. Interestingly, the nucleotide sequence analysis of various genes also indicated differences in their origin. While the identity matrix of VP4 gene (*n* = 3) showed high amino acid sequence identity (97.60 to 98.20%) with Korean strain, the VP6 gene (*n* = 6) showed maximum identity with Nigerian strain (96.40 to 97.60%) and VP7 gene (*n* = 3) with Bangladeshi and USA strains. This is true for all analyzed samples.

**Conclusion:**

Our study demonstrated the group C rotavirus as the cause of severe diarrhea in young children in Delhi and provides insights on the origin of group C rotavirus genes among the local strains indicating their source of transmission. Our study also highlights the need for a simple and reliable diagnostic test that can be utilized to determine the disease burden due to group C rotavirus in India.

## Background

Group C rotavirus (RVC) is an important etiologic agent of dehydrating diarrhea in humans and animals. It belongs to the genus *Rotavirus* within the family *Reoviridae*. Group C Rotaviruses were first detected in piglets with diarrhea in 1980 in USA [1] and their association with the human diarrheal disease was confirmed in 1982 [2]. Thereafter, a number of studies have showed association of group C rotaviruses in many diarrheal outbreaks all over the world [3–7]. The genetic reassortment between porcine RVC and bovine/human RVC were reported earlier [8, 9]. These viruses have also been reported in cases with extrahepatic biliary atresia [10] in which one or more bile ducts are abnormally narrow, blocked or absent. The more vulnerable age for getting group C rotavirus infection is ≥4 years [11] as compared to group A rotavirus where children of less than 3 years of age are at greater risk of getting infected [12, 13].

The prevalence of the group C rotavirus as an etiologic agent of severe diarrhea and the epidemiology of RVC infection is limited due to lack of proper diagnostic assays. The techniques like electron microscopy require a dedicated laboratory set up and cannot be used in a clinical setup for routine detection. Moreover it can identify virus only when present in high concentration and cannot differentiate between group A and group C rotaviruses. Group C rotaviruses are shed in low level and often appear unstable in the stool; therefore RNA may go undetected in the polyacrylamide gel electrophoresis (PAGE). An antigen based Enzyme linked immunosorbant assay (ELISA) using RVC specific antibodies may prove a more sensitive and reliable method for detecting group C rotavirus. The molecular methods like Reverse transcriptase polymerase chain reaction (RT-PCR) with gene specific primers followed by gene sequencing and sequence analysis are more reliable and sensitive tools for the genotyping and characterization of group C rotaviruses [11, 14]. Sequencing followed by phylogenetic analysis of group C viral gene segments offers a better approach for detection of the virus and the investigation of possible evolutionary relationship with other species [15]. This system has provided a reliable platform for determining the origin and comparing the different strains of rotaviruses [16].

The prevalence of group C rotavirus in India remains largely unknown. Therefore the present study was conducted to identify and characterize group C rotavirus in children suffering from acute gastroenteritis admitted in Kalawati Saran Children Hospital (KSCH) in Delhi. RT-PCR followed by sequence analysis was performed to understand its origin and genetic association with other RVC strains reported from different geographical regions globally.

## Methods

### Study sample

A total of 350 fecal specimens were collected between September 2013 and November 2014 from pediatric patients of <5 years admitted with acute gastroenteritis at dedicated child care center “Kalawati Saran Children Hospital” in Delhi as a part of the Rotavirus surveillance study. A written informed consent was obtained from one of the parents of the child before enrollment. All (350) children with diarrhea of less than 3 days duration were screened for group A rotavirus [17]. The remaining group A rotavirus (RV) negative (180) fecal specimens were stored in a deep freezer (−80 °C) for further testing. The study was ethically approved by the institute ethics committee.

### RNA extraction and cDNA synthesis

The dsRNA was extracted from 10% fecal suspension by Trizol method (Invitrogen Corp, Carlsbad, CA) following manufacturer’s instructions. The complementary DNA (cDNA) was synthesized from the RNA using random hexameric primers and moloney murine leukemia virus (M-MLV) reverse transcriptase (Invitrogen Life Technologies). The random priming was carried out by incubation at 37 °C for one hour, followed by 5 min incubation at 95 °C. The cDNA was further utilized for group C VP6, VP7 and VP4 gene amplification.

### PCR Amplification of VP6, VP7 and VP4 genes

Two-step PCR (VP4/VP6/VP7) was carried out, with the first step as full-length gene (VP6/VP7/VP4) amplification with consensus primers (forward and reverse) followed by a nested PCR using the first PCR product and the internal primer(s) [Table 1]. This strategy was adopted to increase the sensitivity of PCR. Further, to reduce the cost of reagents and primers the PCR was carried out first in a pool of 5 cDNA samples (3ul each) and then individual sample from positive pool(s) were retested with gene specific primers [Table 1]. Typically, the PCR mix was prepared containing 2.25ul of 10× buffer II, 2.0ul of 25 mM MgCl_2_, 0.5ul of 10 mM dNTP mix, 0.1ul of 5U/ul Taq polymerase (Invitrogen Life Technologies) and 0.5ul of each 20um/ul of primers to obtain the final reaction volume of 25ul [18]. The PCR was conducted with the condition of initial denaturation at 94 °C for 3 min, 30 cycles of 94 °C for 1 min, 45 °C for 2 min and 72 °C for 3 min and final extension at 72 °C for 7 min. Any change in annealing temperature is specified below. VP6 (1353 bp) full length gene was amplified using VP6 FP-1/BMJ-44. For the nested PCR, BMJ-145 and BMJ-144 were used as internal forward and reverse primer respectively [14, 18]. The nested PCR was carried out at 53 °C annealing temperature [18].

**Table 1:** List of primers used for PCR amplification of group C rotavirus Group C Rotavirus positive controls were provided by Dr. Baoming Jiang, Gastroenteritis & Respiratory Viruses Lab, Division of Viral Diseases, Centers for Disease Control and Prevention, Atlanta

Gene	Primer (Polarity)	Sequence (5′–3′)	Position	Reference
VP7	VP7 FP-1 BMJ-13	5′-GGC ATT TAA AAA AGA AGA AGC TG-3′ 5′-AGC CAC ATG ATC TTG TTT -3′	1–23	Jiang *et al*.,1995
			1063–1046	
	BMJ-107 BMJ-13	5′ TGT TTG GAG ATG TGA TGA -3′ 5′-AGC CAC ATG ATC TTG TTT-3′	546–563	Jiang *et al.,*1995
			1063–1046	
VP6	VP6 FP-1 BMJ-44	5′- GCA TTT AAA ATC TCA TTC ACA A-3′ 5′-AGC CAC ATA GTT CAC ATT TCA-3′	2–22	Jiang *et al.,*1995
			1353–1333	
	BMJ-145 BMJ-144	5′-AGT CCG TTC TAT GTG ATT C-3′ 5′-CCT TCT GGG GAT CAT CCA T-3′	1014–1032	Jiang *et al*.,1995
			1331–1313	
VP4	GCVP4-1FP GCVP4-12RP	5′- GGC TTA AAA AAT AGA GAT CGA TGG CG -3′ 5′- CAT AAA CAA GTT GCA ACC TTG ATG -3′	1–26	Yamamoto *et al*., 2011
			1275–1252	
	GCVP4-2FP GCVP4-12RP	5′- GTA AGG ACT CAT TGT GGC AAG A -3′ 5′- CAT AAA CAA GTT GCA ACC TTG ATG -3′	843–820	Yamamoto *et al*., 2011
			1275–1252	

The full-length VP7 (1063 bp) gene was amplified with VP7 FP-1/BMJ-13 primers. The semi-nested PCR (518 bp) was carried out using the first round product as the template with forward BMJ-107 and reverse BMJ-13 primers [14, 18]. The annealing temperature for VP7 nested PCR was 48 °C. The full-length VP4 gene (1275 bp) was amplified with GCVP4-1FP/GCVP4-12RP primers while for the partial gene (456 bp), GCVP4-2FP and GCVP4-12RP primers were used [9]. The annealing temperature was performed at 52 °C for both first and second round PCR. The primers used for the amplification of VP6, VP7 and VP4 genes are listed in table 1. The PCR products were then analyzed on 2% agarose gel.

### Statistical analysis

The Student t-test was performed to assess the distribution of variables using STRATA version 12. Various clinical parameters such as age, days and episodes of diarrhea and vomiting have been described in terms of mean and standard deviation; the dehydration and treatment variables are presented as percentages.

## Results

### Rotavirus detection

**Fig. 1:**
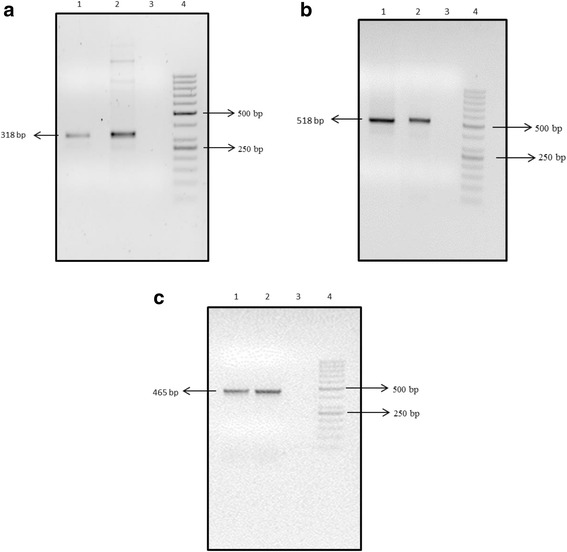
RT- PCR results of group C rotavirus (**a**): VP6 gene (**b**): VP7 gene and **c:** VP4. The amplicons were analyzed on 2% agarose gel stained with ethidium bormide. **a** Lane 1: Sample ND-056, lane 2: positive control, lane 3: negative control and lane 4: 50 bp molecular weight marker. **b** Lane 1: sample ND-061, lane 2: positive control, Lane 3: negative control and lane 4: 50 bp molecular weight marker. **c** Lane 1: Sample ND-061, Lane 2: positive control, lane 3: negative control and lane 4: 50 bp molecular weight marker

A total of 180 of 350 fecal samples previously collected from diarrheal children (<5 years) and tested negative for group A rotavirus (RVA) by Rotaclone ELISA were screened for group C rotavirus by RT-PCR. Six of 180 were found positive for VP6 gene with the prevalence rate of 3.33%. Further 3 out of 6 were found positive for VP4 and VP7 genes [Fig. 1a, b and c].

### Phylogenetic and sequence analysis of VP6 gene

**Fig. 2:**
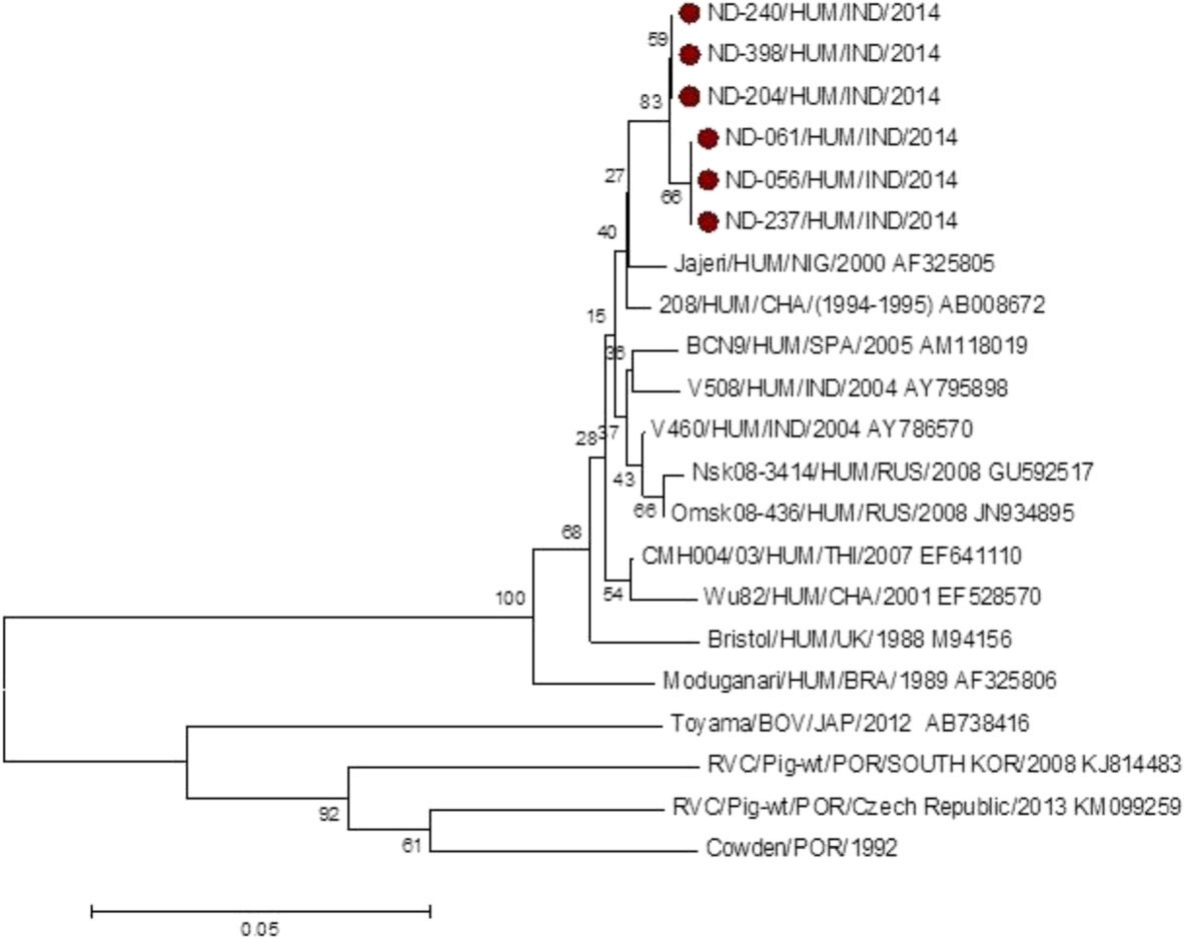
Phylogenetic dendrogram of VP6 gene segment of group C rotavirus by neighbor joining method with MEGA 7.0 version software. The group C positive study samples are indicated by *dark circles*

Phylogenetic analysis of VP6 gene (*n* = 6) with reference strains showed clustering with Nigerian and Chinese human RVC [Fig. 2]. A maximum homology both at nucleotide (98.6 to 99.7%) as well as amino acid (96.4 to 97.60%) level was observed with the Nigerian strain ‘jajeri’ and relatively less homology (90.60 to 93.00% nucleotide and 95.10 to 96.60% for amino acid) was observed with previously reported Indian strains [Table 2]. Overall high VP6 sequence homology was generally seen with most human RVC strains as compared to those of animal rotavirus strains. For example: 92.10 to 96.20% (amino acid) homology was observed with other human strains as compared to 60.20 to 67.60% (amino acid) homology with bovine and porcine strains respectively. Among the study sample, ND-204, ND-240 and ND-398; ND-056, ND-061 and ND-237 were found identical with respect to VP6 gene [Table 2].

**Table 2:** Percentage identity matrices (nucleotide and deduced amino acid) of VP6 gene

Strain			Human				Bovine	Porcine	Human/India		Study Samples					
	Bristol	BCN9	OH567	Wu82	BS347	Jajeri	Toyama	RVC	V460	V508	ND-056	ND-061	ND-204	ND-237	ND-240	ND-398
Bristol		92.10%	96.90%	92.10%	96.90%	92.40%	79.00%	79.30%	92.40%	92.10%	99.00%	99.00%	98.70%	99.00%	98.70%	98.70%
BCN9	97.80%		92.70%	97.60%	93.20%	98.50%	84.80%	84.20%	99.10%	98.80%	96.70%	96.70%	97.70%	96.70%	97.70%	97.70%
OH567	92.10%	90.60%		93.80%	97.50%	93.00%	78.70%	79.30%	93.50%	92.70%	95.30%	95.30%	96.30%	95.30%	96.30%	96.30%
Wu82	96.70%	93.30%	94.20%		92.10%	97.90%	84.50%	84.50%	98.50%	97.60%	95.30%	95.30%	97.10%	95.30%	97.10%	97.10%
BS347	91.10%	90.50%	93.00%	97.60%		93.00%	79.80%	79.80%	93.50%	93.20%	96.90%	96.90%	96.90%	96.90%	96.90%	96.90%
Jajeri	92.60%	95.20%	90.40%	94.20%	92.50%		84.80%	84.80%	98.80%	98.50%	98.60%	98.60%	99.70%	98.60%	99.70%	99.70%
Toyama	68.60%	67.10%	62.30%	66.10%	61.40%	66.10%		88.90%	84.80%	85.10%	77.00%	77.00%	78.70%	77.00%	78.70%	78.70%
RVC	66.60%	67.10%	60.40%	64.20%	61.40%	66.10%	88.00%		84.80%	83.90%	77.00%	77.00%	78.70%	77.00%	78.70%	78.70%
V460	95.30%	94.80%	95.30%	92.90%	91.10%	95.30%	64.80%	64.80%		99.10%	90.90%	90.90%	93.00%	90.90%	93.00%	93.00%
V508	96.20%	92.00%	96.20%	91.60%	95.80%	93.50%	63.50%	61.60%	92.90%		90.60%	90.60%	92.70%	90.60%	92.70%	92.70%
ND-056	96.20%	95.10%	96.00%	95.70%	96.20%	97.60%	67.60%	66.60%	96.40%	95.10%		100.00%	98.60%	100.00%	98.60%	98.60%
ND-061	96.20%	95.10%	96.00%	95.70%	96.20%	97.60%	67.60%	66.60%	96.40%	95.10%	100.00%		98.60%	100.00%	98.60%	98.60%
ND-204	95.40%	93.20%	93.20%	95.30%	92.10%	96.40%	61.40%	60.20%	95.20%	96.60%	97.20%	97.20%		97.20%	100.00%	100.00%
ND-237	96.20%	95.10%	96.00%	95.70%	96.20%	97.60%	67.60%	66.60%	96.40%	95.10%	100.00%	100.00%	98.60%		98.60%	97.20%
ND-240	95.40%	93.20%	93.20%	95.30%	92.10%	96.40%	61.40%	60.20%	95.20%	96.60%	97.20%	97.20%	100.00%	97.20%		100.00%
ND-398	95.40%	93.20%	93.20%	95.30%	92.10%	96.40%	61.40%	60.20%	95.20%	96.60%	97.20%	97.20%	100.00%	97.20%	100.00%	

Identity matrices of the nucleotide (above the diagonal) and deduced amino acid (below the diagonal) among human, porcine and bovine group C rotavirus strains. Reference strains from different geographical locations are used in the analysis

### Phylogenetic and sequence analysis of VP7 gene

**Fig. 3:**
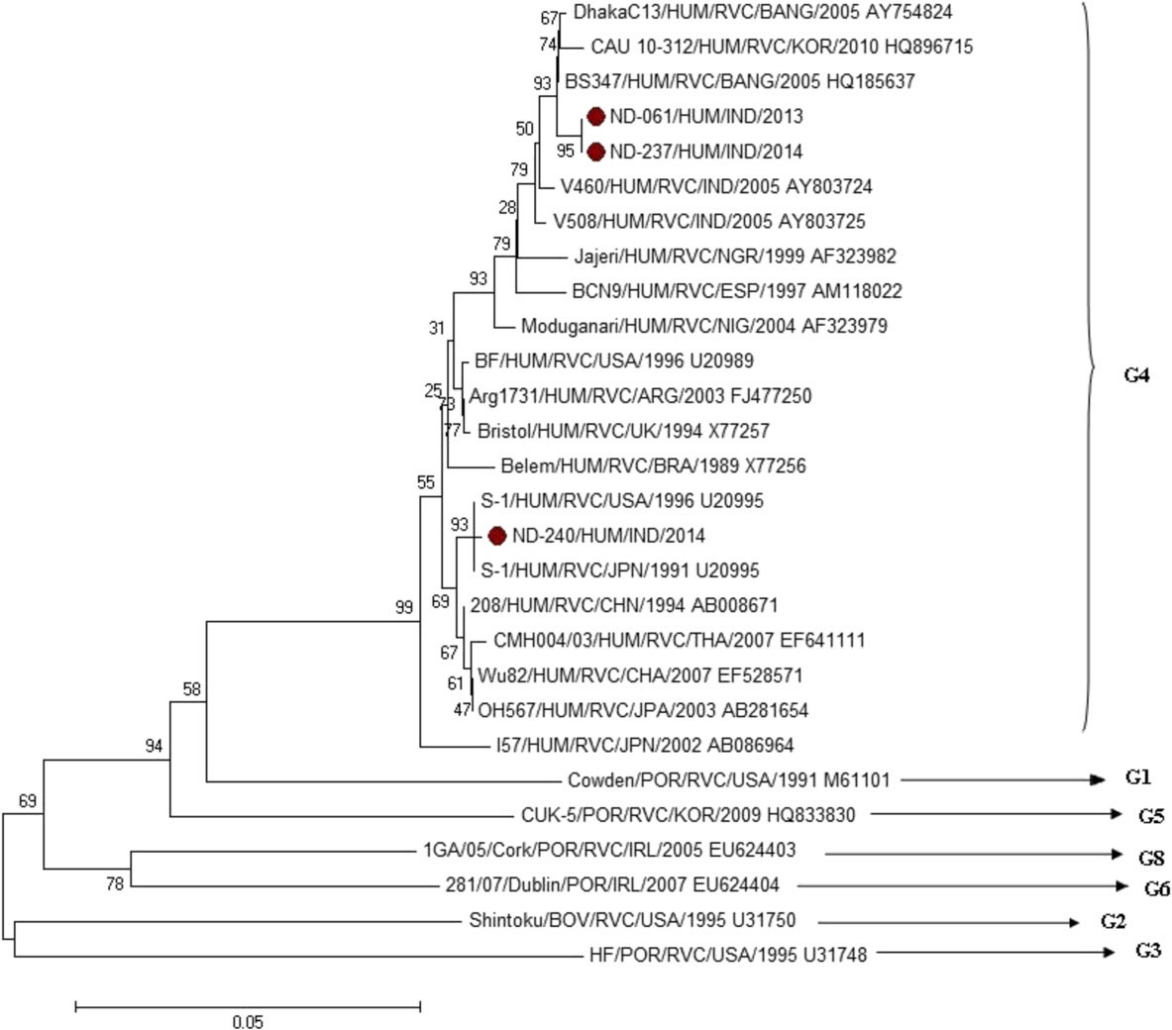
Phylogenetic dendrogram of VP7 gene segment of group C rotavirus by neighbor joining method with MEGA 7.0 version software. The group C positive study samples are indicated by *dark circles*

The phylogenetic dendrogram of VP7 showed the infecting RVC strains (*n* = 3) clustering into a single genotype “G4”, which is the only human RVC genotype. The ND-240 strain showed close genetic relationship with USA strain while ND-061 and ND-237 clustered with Bangladeshi human RVC strains [Fig. 3]. The VP7 gene of the present RVC strains revealed 98.0 to 99.20% at nucleotide and 95.70 to 97.60% at amino acid level with Bangladeshi and USA strains [Table 3]. However, relatively less homology (96.90 to 97.80% nucleotide and 90.90 to 97.90% amino acid) was observed with Indian strains reported earlier [Table 3]. The animal RVC such as bovine (73.20 to 73.80% amino acid) and porcine (75.00 to 75.60% amino acid) strains were found distantly related. The VP7 sequence analysis of our study sample showed 98.20 to 100.0% nucleotide identity with each other.

**Table 3:** Percentage identity matrices (nucleotide and deduced amino acid) of VP7

Strain					Human			Porcine	Bovine	Human/India		Study Samples		
	Bristol	BCN9	OH567	Wu82	BS347	C13	S-1	Cowden	Shintoku	V460	V508	ND-061	ND-240	ND-237
Bristol		96.70%	98.20%	98.20%	96.70%	96.30%	98.20%	75.10%	79.00%	96.70%	96.70%	97.10%	98.40%	97.10%
BCN9	90.70%		95.30%	95.30%	97.30%	96.90%	95.30%	74.20%	78.60%	97.60%	97.60%	97.70%	98.90%	97.70%
OH567	95.00%	95.80%		100.00%	95.70%	95.30%	98.80%	75.50%	79.40%	95.70%	96.10%	96.20%	97.40%	96.20%
Wu82	95.00%	93.80%	100.00%		95.70%	95.30%	98.80%	75.50%	79.40%	95.70%	96.10%	96.20%	97.00%	96.20%
BS347	91.90%	93.80%	91.50%	93.30%		99.60%	96.10%	75.00%	78.40%	99.20%	98.40%	98.40%	98.60%	98.40%
C13	90.70%	92.60%	93.30%	91.50%	98.70%		95.70%	74.60%	78.00%	98.80%	98.00%	98.00%	98.20%	98.00%
S-1	95.00%	95.80%	96.20%	96.20%	90.70%	94.50%		75.70%	79.80%	96.10%	96.10%	99.20%	94.80%	99.20%
Cowden	70.70%	73.80%	78.20%	78.20%	76.70%	75.10%	76.50%		79.40%	75.10%	75.10%	73.00%	74.10%	73.00%
Shintoku	71.30%	74.40%	70.00%	79.10%	76.90%	75.70%	70.00%	76.90%		78.80%	78.20%	77.80%	78.40%	77.80%
V460	94.80%	92.50%	95.40%	95.40%	94.40%	94.00%	96.00%	75.00%	75.40%		98.80%	97.60%	97.80%	97.60%
V508	95.10%	97.50%	96.70%	95.70%	97.10%	97.80%	98.30%	77.10%	76.70%	98.70%		96.90%	97.10%	96.90%
ND-061	95.50%	97.40%	96.40%	96.40%	96.90%	95.70%	97.60%	75.60%	73.80%	92.50%	90.90%		98.20%	100.00%
ND-240	98.80%	97.70%	95.70%	95.80%	95.10%	96.20%	97.00%	75.00%	73.20%	97.90%	96.50%	96.20%		98.20%
ND-237	95.50%	97.40%	96.40%	96.40%	96.90%	95.70%	97.60%	75.60%	73.80%	92.50%	90.90%	100.00%	96.50%	

Identity matrices nucleotide (above the diagonal) and deduced amino acid (below the diagonal) among human, porcine and bovine group C rotaviruses. References strains from different geographical locations are used for the analysis

### Phylogenetic and sequence analysis of VP4 gene

**Fig. 4:**
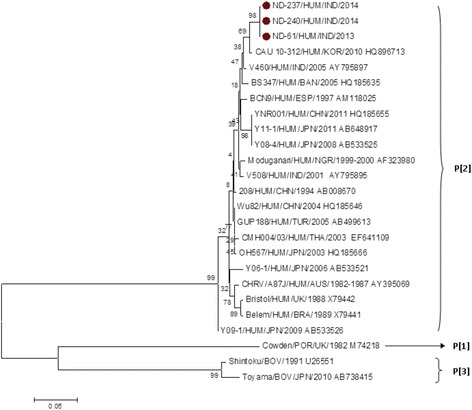
Phylogenetic dendrogram of VP4 gene segment of group C rotavirus by neighbor joining method with MEGA 6.0 version software. The group C VP4 positive samples of our study are indicated by *dark circles*

The VP4 genes of the present RVC strains clustered with P[2] genotype which is the only known human P genotype [Fig. 4]. A close VP4 sequence homology was observed with Korean strain “CAU 10-312” both at nucleotide (98.70 to 99.50%) and amino acid (97.60 to 98.20%) level [Table 4]. However, relatively less homology (nucleotide identity of 97.30 to 98.10% and amino acid identity 96.30 to 97.90%) was observed with previously reported Indian strains. Similar to the VP6 and VP7 genes the VP4 gene of animal RVC were also found distantly related to the present RVC; for example amino acid identity of 66.80 to 67.40% and 68.10 to 68.70% were observed with bovine and porcine strains respectively [Table 4]. Of note, VP4 gene of ND-061 and ND-237 were also found identical as seen in case of VP7.

**Table 4:** Percentage identity matrices (nucleotide and deduced amino acid) of VP4 gene

Strains			Human				Porcine	Bovine	Human/India		Study Samples		
	Bristol	BCN9	OH567	Wu82	BS347	CAU 10–312	Cowden	Shintoku	V460	V508	ND-061	ND-240	ND-237
Bristol		97.00%	97.00%	97.60%	96.10%	96.50%	69.20%	65.20%	96.60%	97.00%	98.40%	99.90%	98.40%
BCN9	91.90%		97.80%	98.50%	97.80%	97.40%	68.80%	64.40%	98.30%	98.70%	97.90%	98.20%	97.90%
OH567	91.90%	94.60%		99.30%	97.00%	97.00%	69.00%	65.00%	97.40%	98.20%	98.20%	98.40%	98.20%
Wu82	93.20%	95.90%	98.60%		97.60%	97.60%	69.20%	65.00%	98.00%	98.50%	97.70%	98.90%	97.70%
BS347	89.90%	93.90%	92.60%	93.90%		97.40%	68.80%	64.40%	97.80%	97.80%	97.10%	98.40%	97.10%
CAU 10–312	91.20%	92.60%	92.60%	93.90%	93.20%		69.20%	65.20%	97.80%	97.40%	98.70%	99.50%	98.70%
Cowden	69.70%	69.10%	69.70%	69.70%	67.20%	60.30%		75.70%	69.00%	69.20%	66.80%	67.80%	66.80%
Shintoku	69.00%	67.80%	69.00%	69.00%	67.80%	69.60%	77.30%		64.60%	64.80%	63.20%	63.60%	63.20%
V460	90.60%	94.60%	93.30%	94.60%	94.00%	94.00%	69.10%	67.80%		98.30%	97.30%	98.00%	97.30%
V508	92.60%	96.60%	95.30%	96.60%	94.60%	93.20%	69.10%	67.80%	95.30%		98.10%	97.40%	98.10%
ND-061	92.50%	95.90%	94.50%	95.90%	95.90%	98.20%	68.10%	66.80%	97.30%	96.50%		97.20%	100.00%
ND-240	93.80%	97.20%	95.90%	97.20%	97.20%	97.60%	68.70%	67.40%	96.30%	97.90%	99.50%		98.20%
ND-237	92.50%	95.90%	94.50%	95.90%	95.90%	98.20%	68.10%	66.80%	97.30%	96.50%	100.00%	99.50%	

Identity matrices nucleotide (above the diagonal) and deduced amino acid (below the diagonal) among human, porcine and bovine group C rotaviruses. References strains from different geographical locations are used for the analysis

For all the three genes VP6, VP7 and VP4, both bovine and porcine strains formed the two separate cluster groups and had poor homology with the human strains [Table 5].

**Table 5:** Comparative sequence identities (%) of the rotavirus group C study strains with that of human, bovine and porcine strains from other studies

	Human Strains		Bovine		Porcine	
Gene	Nucleotide	Amino acid	Nucleotide	Amino acid	Nucleotide	Amino acid
VP4	96.10–100	89.90–100	63.20–65.20	66.80–69.60	66.80–69.20	60.30–69.70
VP6	90.60–100	90.40–100	77.0–85.80	61.40–68.60	77.0–84.80	60.20–67.10
VP7	94.10–100	91.50–100	77.8–79.4	70.0–79.1	83.80–85.50	70.70–78.20

The human strains include Bristol, BCN9, BS347, OH567, Wu82, V508 and V460, ND-061, ND-204, ND-398, ND-237, ND-056, ND-240

Table 6 describes the clinical details of the RVC negatives (including group A positives) and RVC positive patients. Our data indicated that the boys were at higher risk of getting RVC infection than girls. The dehydration was found more severe in RVC positive patients than in RVC negative patients. However, this difference was not statistically significant. Other clinical parameters like age, duration of diarrhea, vomiting days and episodes and treatment are similar in both groups of patients [Table 6].

**Table 6:** Comparison of demographic and clinical characteristics of children having acute RVC diarrhea with that of unknown etiology

Parameters	RVC negative	RVC positive	
	patients (*n* = 344)	patients (*n* = 6)	
Gender (Male/Female)	64.8% / 35.17%	66.7% / 33.3%	
Age (mean ± SD) (months)	12.39 (±8.41)	17 (±11.15)	*p* (0.09)
Days of Diarrhea (mean ± SD)	2.85 ± 1.95	4.33 ± 2.94	*p* (0.06)
Episodes (mean ± SD)	16.08 ± 8.05	20.67 ± 10.72	*p* (0.16)
Severe Dehydration	72.20%	100%	
Days of vomiting (mean ± SD)	1.95 ± 1.70	1 ± 1.26	*p* (0.17)
Episodes/day (mean ± SD)	5.26 ± 4.26	4.5 ± 5.92	*p* (0.67)
Treatment (Oral/Intravenous)	24.7% / 75.29%	16.6% / 83.3%	

## Discussion

**Fig. 5:**
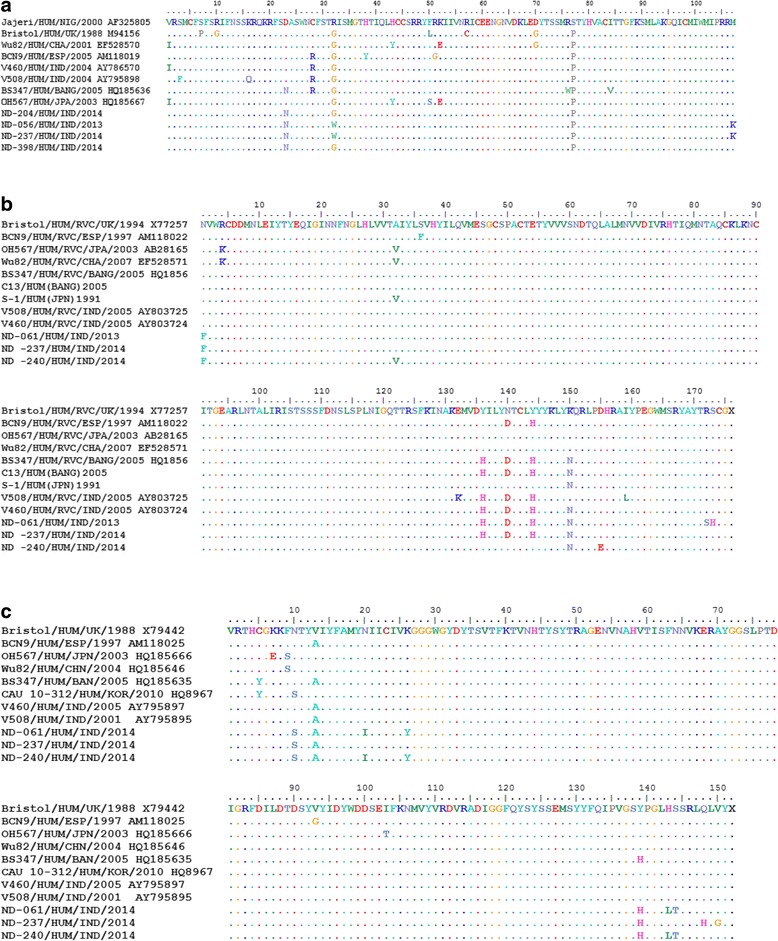
Deduced amino acid sequence alignment of (**a**): VP6, (**b**): VP7 and (**c**): VP4 genes of previous Indian RVC strains and strains from our study. The *dots* represent the identical amino acids

Since the first outbreak of group C rotavirus in the year 1980, in USA, there have been various outbreaks reported from different parts of the world. The serosurveillance study conducted in Sweden reported group C prevalence ranging from 35 to 45% depending on age [19]. A survey in UK reported the group C seroprevalence of 43% in all age groups and 66% for 71–75 years age group [20]. The detection of group C rotavirus and its prevalence has been extensively studied in many countries worldwide. However, its prevalence in India remains largely unknown. Very recently around the same time as our study, another study was conducted in Western India also detected RVC in a group of patients aged 9 months to 86 years [21]. The present study for the first time is conducted in Northern India that identifies group C rotavirus as the etiological agent of severe diarrhea in children in Delhi. Jiang et al. in [22] and Martella et al. in [23] proposed a classification of RVC and accordingly group C rotaviruses were further classified into G (VP7) and P (VP4) genotypes similar to group A rotavirus [22, 23]. Earlier studies had shown a total of 9 ‘G’ and 3 ‘P’ genotypes of RVC including humans and animals strains [9, 20–25]. All previous reports (Jiang et al. in [22], Martell et al. in [23] and Luchs et al. in [26]) showed that the human RVC strains fall into a single ‘G’ (G4) and single ‘P’ (P[2]) genotype. On the contrary, animal RVC strains fall into multiple G/P types. For example, bovine strains are G2P[3] and porcine strains are G1,G3,G5,G6;P[1] genotypes [22, 23, 26]. All earlier studies conducted worldwide had demonstrated the phylogenetic relatedness among human RVC strains [27–30]. Our results further confirmed the earlier reports including that of Yamamoto D et al. and Luchs et al. on human group C rotavirus VP7 and VP4 genes being highly conserved clustering to G4P[2] genotype [9, 25]. Additionally our nucleotide and phylogenetic data showed that VP4, VP6 and VP7 genes were more closely related within themselves as contrary to what has been reported earlier. This may be attributed to the different geographical locations. However, according to Khamrin et al., group C rotavirus strains from animals remain different from the human RVC strains [31]. The differences in sequence homology with respect to VP7 (close to Bangladeshi and USA strains) and VP6 (close to Nigerian and Chinese strain) genes clearly showed differences in their origin. The phylogenetic dendrogram of VP6 gene of ND isolates showed higher sequence homology with Nigerian strain “jajeri” and Chinese strain “208” as compared to previously reported Indian strains indicating difference in emergence. The VP7 gene analysis further illustrated that the study strains fall into two different groups, one was found to be more closely related to Bangladeshi strain “Dhaka C13” while the second group was found clustering with USA strains suggesting two separate sources of origin. The molecular data of animal and human group C rotaviruses will help to further elucidate the origin of human RVC. As anticipated, substitutions in the deduced amino acid sequences were more variable in VP7 and VP4 as compared to VP6 sequences [Figs. 5a, b and c]. This indicates that the genes VP7 and VP4 have evolved more over the period of time from the year 2001 to 2014 whereas the evolution or genetic drift in VP6 gene is slow.

RVCs have been found as important enteric pathogens because they cause diarrhea in all age groups, including adults, [32–34]. However, RVC are mostly detected in children above 4 years of age [11, 14]. Our study also detected group C rotavirus in children from 3 months to 2.8 years age group. This shows that the children less than 4 years old may be at greater risk of getting infected with RVC.

Some of the group C VP6 positive sample in the present study could not be amplified with the VP4 and VP7 genotyping primers. This can be attributed to sequence difference(s) in the primer binding region. Luchs and Timenetsky in [26] also reported non-typeable strains of group C rotavirus in Brazil [25]. Sequence analysis of other RV genes from these samples is in progress which will help us to understand their diversity and elucidate their origin. Given that RVC often appear unstable, screening of larger number of samples will help to determine the true estimate of disease burden due to RVC in India.

Our study emphasizes the need to conduct more surveillance studies and detailed investigations on group C rotavirus, initially in the sentinel sites that can be further extended to other geographical locations in India. This will help in estimating the actual load of gastroenteritis in Indian population and will also help to understand whether the new Indian rotavirus vaccine (Rotavac®) has the ability to cross protect other groups of rotaviruses in humans.

## Conclusion

Our study demonstrated group C rotavirus as the cause of severe diarrhea in children in Delhi and emphasizes the need to determine the disease burden using a sensitive and reliable diagnostic test.

## Abbreviations

PAGE: Polyacrylamide gel electrophoresis
RT-PCR: Reverse transcription polymerase chain reaction
RVC: Group C rotavirus
